# Structural and functional characterization of MERS coronavirus papain-like protease

**DOI:** 10.1186/1423-0127-21-54

**Published:** 2014-06-04

**Authors:** Min-Han Lin, Shang-Ju Chuang, Chiao-Che Chen, Shu-Chun Cheng, Kai-Wen Cheng, Chao-Hsiung Lin, Chiao-Yin Sun, Chi-Yuan Chou

**Affiliations:** 1 Department of Life Sciences and Institute of Genome Sciences, National Yang-Ming University, Taipei 112, Taiwan; 2 Department of Nephrology, Chang-Gung Memorial Hospital, Keelung 204, Taiwan

**Keywords:** MERS coronavirus, Papain-like protease, Deubiquitination, Antiviral target

## Abstract

**Backgrounds:**

A new highly pathogenic human coronavirus (CoV), Middle East respiratory syndrome coronavirus (MERS-CoV), has emerged in Jeddah and Saudi Arabia and quickly spread to some European countries since September 2012. Until 15 May 2014, it has infected at least 572 people with a fatality rate of about 30% globally. Studies to understand the virus and to develop antiviral drugs or therapy are necessary and urgent. In the present study, MERS-CoV papain-like protease (PL^pro^) is expressed, and its structural and functional consequences are elucidated.

**Results:**

Circular dichroism and Tyr/Trp fluorescence analyses indicated that the secondary and tertiary structure of MERS-CoV PL^pro^ is well organized and folded. Analytical ultracentrifugation analyses demonstrated that MERS-CoV PL^pro^ is a monomer in solution. The steady-state kinetic and deubiquitination activity assays indicated that MERS-CoV PL^pro^ exhibits potent deubiquitination activity but lower proteolytic activity, compared with SARS-CoV PL^pro^. A natural mutation, Leu105, is the major reason for this difference.

**Conclusions:**

Overall, MERS-CoV PL^pro^ bound by an endogenous metal ion shows a folded structure and potent proteolytic and deubiquitination activity. These findings provide important insights into the structural and functional properties of coronaviral PL^pro^ family, which is applicable to develop strategies inhibiting PL^pro^ against highly pathogenic coronaviruses.

## Background

In September 2012, a new highly pathogenic human coronavirus (CoV)^1^, Middle East respiratory syndrome coronavirus (MERS-CoV), has emerged in Jeddah and Saudi Arabia and quickly spread to some European countries [1-3]. The virus causes symptoms similar to Severe Acute Respiratory Syndrome Coronavirus (SARS-CoV), yet involving an additional component of acute renal failure [4]. Until 15 May 2014, it has infected at least 572 people with a fatality rate of about 30% globally (World Health Organization, global alert and response, http://www.who.int/csr/don/2014_05_15_mers/en/). Recently, human-to-human transmission of MERS-CoV has been confirmed; albeit, a serological study of major livestock suggested dromedary camels also to be a possible host [5,6]. Nevertheless, these findings indicate that the virus have the opportunity to spread globally and pose a significant threat to world health and the economy. Therefore, studies to understand the virus and to develop antiviral drugs or therapy are necessary and urgent.

Like other CoVs, the MERS-CoV nonstructural polyproteins (pp1a and pp1ab) are cleaved by two types of viral cysteine proteases, a main protease (EC 3.4.22.69) and a papain-like protease (PL^pro^) (EC 3.4.22.46) [7]. This processing is considered to be a suitable antiviral target because it is required for viral maturation. Unfortunately, initial screening of the existing SARS-CoV PL^pro^ inhibitor, a benzodioxolane derivative against MERS-CoV PL^pro^, revealed no significant inhibition [7]. The difference represents the requirement of further understanding the MERS-CoV PL^pro^. In addition to proteolytic activity, similar to those of SARS-CoV, NL63-CoV and murine hepatitis virus, MERS-CoV PL^pro^ acts on both deubiquitination and ISG15-linked ISGylation [8-11]. As a viral deubiquitinating protease (DUB), MERS-CoV PL^pro^ is able to deubiquitinate interferon regulatory factor 3 (IRF3), which can prevent its nuclear translocation and suppress production of interferon β [10]. These studies support the multifunctional nature of coronaviral PL^pro^. Recently, with the crystal structure of SARS-CoV PL^pro^ C112S mutant in complex with ubiquitin (Ub), we have demonstrated that Ub core (residue 1–72) makes mostly hydrophilic interactions with PL^pro^, while the Leu-Arg-Gly-Gly C-terminus of Ub is located in the catalytic cleft of PL^pro^, mimicking the P4-P1 residues [12]. This bound pattern is similar to that of the ubiquitin-specific proteases (USPs), one of the five distinct DUB families [13,14].

The MERS-CoV PL^pro^ domain in nsp3 of the pp1a proteins (residue 1484–1800) has been identified [7,10,15]. Like other PL^pro^, there is a catalytic triad consisting of the residues Cys1592, His1759 and Asp1774. Homology modeling suggests that MERS-CoV PL^pro^, similar to other known PL^pro^, may have a right-hand-like architecture constituted by palm, thumb, and fingers domains, although their sequence identity are only about 30% [12]. Furthermore, MERS-CoV PL^pro^ is able to recognize and cleave at the LXGG consensus cleavage site, which is essential for most CoV PL^pro^-mediated processing [10]. Despite this large body of knowledge on MERS-CoV PL^pro^, in the absence of detailed structural and functional characterization, the molecular basis for its catalytic mechanism remains poorly unknown.

Here, we expressed and purified the MERS-CoV PL^pro^ by *E. coli* with high yield and high purity. The secondary, tertiary and quaternary structure of MERS-CoV PL^pro^ was then investigated by circular dichroism (CD) spectroscopy, Tyr/Trp fluoresecence and analytical ultracentrifugation (AUC), respectively. The kinetic and DUB activity assays indicated that MERS-CoV PL^pro^ exhibits potent DUB activity but lower proteolytic activity, compared with SARS-CoV PL^pro^. The present study provides a foundation for understanding the structural and biochemical properties of coronaviral PL^pro^ family, which is applicable to develop strategies inhibiting PL^pro^ for the effective control of highly pathogenic coronaviral infection.

## Methods

### Expression plasmid construction

The sequence of MERS-CoV PL^pro^ (GenBank accession number NC_019843.2; polyprotein residues 1484–1800) was synthesized (MDBio Inc.), digested by *Nco*I-*Xho*I and then inserted into the pET-28a(+) vector (Novagen). In the construct, the 6 x His tag was retained at the C-terminus. The reading frame was confirmed by sequencing.

### Expression and purification of MERS-CoV PL^pro^

The expression vector was transformed into *E. coli* BL21 (DE3) cells (Novagen). For large scaled protein expression, cultures were grown in LB medium of 0.8 liter at 37°C for 4 h, induced with 0.4 mM isopropyl-β-_D_-thiogalactopyranoside, and incubated overnight at 20°C. After centrifuging at 6,000 x g at 4°C for 15 min, the cell pellets were resuspended in lysis buffer (20 mM Tris, pH 8.5, 250 mM NaCl, 5% glycerol, 0.2% Triton X-100, and 2 mM β-mercaptoethanol) and then lysed by sonication. The crude extract was then centrifuged at 12,000 x g at 4°C for 25 min to remove the insoluble pellet. The supernatant was incubated with 1-ml Ni-NTA beads at 4°C for 1 h and then loaded into an empty column. After allowing the supernatant to flow through, the beads were washed with washing buffer (20 mM Tris, pH 8.5, 250 mM NaCl, 8 mM imidazole, and 2 mM β-mercaptoethanol), and the protein was eluted with elution buffer (20 mM Tris, pH 8.5, 30 mM NaCl, 150 mM imidazole, and 2 mM β-mercaptoethanol). The protein was then loaded onto a S-100 gel-filtration column (GE Healthcare) equilibrated with running buffer (20 mM Tris, pH 8.5, 100 mM NaCl, and 2 mM dithiothreitol). The purity of the fractions collected was analyzed by SDS-PAGE and the protein was concentrated to 30 mg/ml by Amicon Ultra-4 10-kDa centrifugal filter (Millipore).

### Circular dichroism spectroscopy

CD spectra of the recombinant MERS-CoV PL^pro^ using a JASCO J-810 spectropolarimeter showed measurements from 250 to 190 nm at 20°C in 50 mM phosphate pH 6.5. The protein concentration was 1.0 mg/ml. In wavelength scanning, the width of the cuvette was 0.1 mm. The far-UV CD spectrum data were analyzed with the CDSSTR program [16,17]. In this analysis, the α-helix, β-sheet, and random coil were split. To estimate the goodness-of-fit, the normalized root mean square deviation was calculated.

### Spectrofluorimetric analysis

The fluorescence spectra of the enzyme at 1 μM were monitored in a Perkin-Elmer LS50B luminescence spectrometer at 25°C. The excitation wavelength was set at 280 nm, and the fluorescence emission spectrum was scanned from 300 to 400 nm. Measurement in the maximal peak, intensity, and average emission wavelength were used to confirm the protein folding [18,19].

### Analytical ultracentrifugation analysis

The AUC experiments were performed on a XL-A analytical ultracentrifuge (Beckman Coulter) using an An-50 Ti rotor [12,19-22]. The sedimentation velocity experiments were performed using a double-sector *epon* charcoal-filled centerpiece at 20°C with a rotor speed of 42,000 rpm. Protein solutions of MERS-CoV PL^pro^ (1.0 mg/ml) (330 μl) and reference (370 μl) solutions were loaded into the centerpiece, respectively. The absorbance at 280 nm was monitored in a continuous mode with a time interval of 300 s and a step size of 0.003 cm. Multiple scans at different time intervals were then fitted to a continuous c(s) distribution model using the SEDFIT program [23]. All size-and-shape distributions were analyzed at a confidence level of p = 0.95 by maximal entropy regularization and a resolution N of 200 with sedimentation coefficients between 0 and 20 S or molar mass between 0 and 1000 kDa.

### Steady-state kinetic analysis

The peptidyl substrate, Dabcyl–FRLKGGAPIKGV–Edans, was used to measure the enzymatic activity of MERS-CoV PL^pro^ and its mutants throughout the course of the study as described [24]. Specifically, the enhanced fluorescence emission upon substrate cleavage was monitored at excitation and emission wavelengths of 329 and 520 nm, respectively, in a PerkinElmer LS 50B luminescence spectrometer. Fluorescence intensity was converted to the amount of hydrolyzed substrate using a standard curve drawn from the fluorescence measurements of well-defined concentrations of Dabcyl–FRLKGG and APIKGV–Edans peptides in a 1:1 ratio. This will also correct for the inner filter effect of the substrate. For the kinetic analysis, the reaction mixture contained 4–50 μM peptide substrate in 50 mM phosphate pH 6.5 in a total volume of 1 mL. After the addition of the enzyme to the reaction mixture, the increase in fluorescence was continuously monitored at 30°C. The increase in fluorescence was linear for at least 3 min, and thus the slope of the line represented the initial velocity (*v*). The steady-state kinetic parameters of the enzyme were determined by fitting the Michaelis–Menten equation (eq. 1) to the initial velocity data

(1) $$v=\frac{k_{\mathrm{cat}}\left[E\right]\left[S\right]}{K_{m}+\left[S\right]}$$

in which *k*_cat_ is the rate constant, [*E*] and [*S*] denote the enzyme and substrate concentration, and *K*_m_ is the Michaelis-Menten constant for the interaction between the peptide substrate and the enzyme.

### Deubiquitination assay

The fluorogenic substrate Ub-7-amino-4-trifluoro-methylcoumarin (Ub-AFC) (Boston Biochem) added at 0.5 or 1.0 μM to 50 mM phosphate pH 6.5 was used for deubiquitination assays as described [12]. The enzymatic activity at 30°C was determined by continuously monitoring the fluorescence emission and excitation wavelength of 350 and 485 nm, respectively.

## Results and discussion

### Recombinant MERS-CoV PL^pro^ preparation

**Figure 1:**
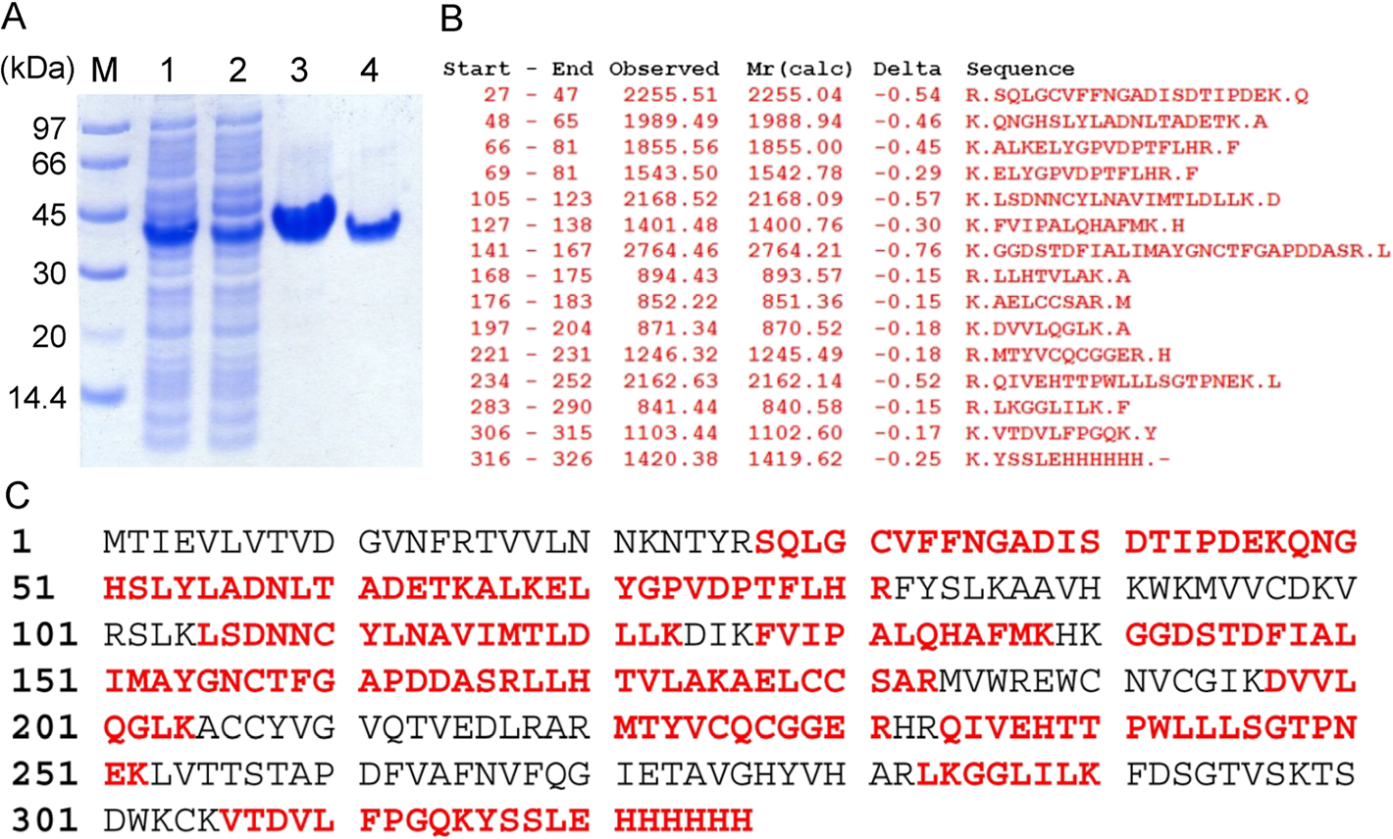
**Expression and purification of recombinant MERS**-**CoV PL**^**pro**^**. (A)** Protein identification by SDS-PAGE. M: molecular marker. Lane 1–4: cytoplasmic fraction, flow-through, elution from the nickel affinity column and protein fraction from S-100 gel-filtration column. **(B)** and **(C)** Protein sequence identification by mass spectrometry. The PL^pro^ was digested by trypsin and then analyzed by MALDI mass spectrometry. There are 15 matched peptides observed **(B)** and 60% sequence coverage are shown in bold red **(C)**.

To date, there are still no studies describing the expression and purification of MERS-CoV PL^pro^ proteins. In the present study, the expression vector was constructed and then various *E. coli*. strains such as BL21 (DE3) STAR (Invitrogen) and Rosetta (DE3) (Novagen) were used to explore heterologous expression of MERS-CoV PL^pro^. Finally, it was found that the STAR strain showed the best expression efficiency. After expressing the protein in *E. coli* and purification by nickel affinity chromatography and gel-filtration, the purity of recombinant PL^pro^ was about 99% (Figure 1A). The size of the recombinant MERS-CoV PL^pro^ was found to be between 30 and 45 kDa, which conforms to the theoretical mass (36.5 kDa). The typical yield was about 42 mg after purification from 0.8 liter of *E. coli* culture (Table 1). After gel-filtration chromatography, the specific proteolytic activity of PL^pro^ was 4 U/mg, increased by 5-fold, with 49.4% recovery rate.

**Table 1:** **Purification of MERS**-**CoV PL**^**pro **^**from *E. coli***

**Step**	**Total protein (mg)**	**Total activity (U**^**a**^**)**	**Specific activity (U**/**mg protein)**	**Purification (**-**fold)**	**Recovery (%)**
Cytoplasmic fraction	426	340	0.80	1	100
Ni affinity chromatography	64.4	221	3.43	4.3	65
Gel-filtration by S-100 column	41.9	168	4.01	5.0	49.4

^a^One unit is defined as the amount of enzyme required to catalyze the cleavage of 1 nmole of peptidyl substrate (Dabcyl-FRLKGGAPIKGV-Edans) per minute at 30°C.

Furthermore, the recombinant MERS-CoV PL^pro^ was digested by trypsin and then analyzed by MALDI mass spectrometry to confirm the amino acid sequence (Additional file 1: Figure S1). The molecular weight of fifteen peptides, which covered 60% amino acid sequence, was observed and confirmed (Figure 1B and Figure 1C). It indicated that our expression and purification of MERS-CoV PL^pro^ by *E. coli* is successful. For convenience, in the present studies, the MERS-CoV PL^pro^ domain (polyprotein 1a 1484–1800) is numbered to residue 2 to 317, while the first residue is a methionine.

### Secondary, tertiary and quaternary structure analysis of MERS-CoV PL^pro^

**Figure 2:**
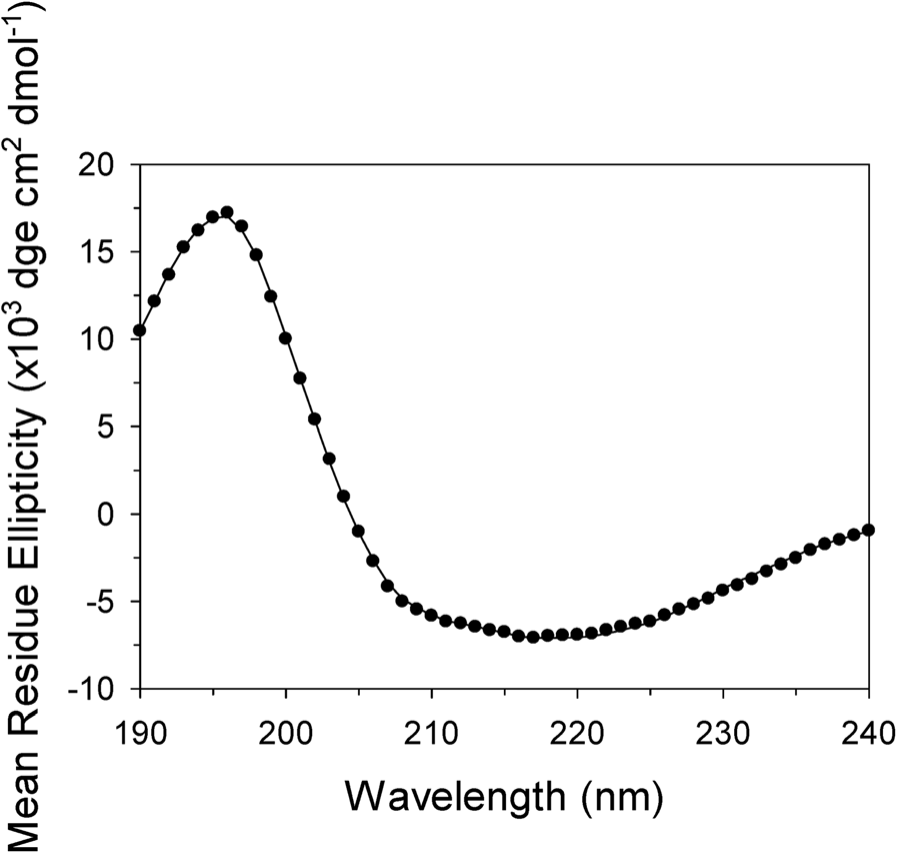
**CD spectrum of MERS**-**CoV PL**^**pro**^**.** The protein of 1 mg/ml was suspended in 50 mM phosphate pH 6.5 and the CD values were measured from 240 to 190 nm at 20°C. The obtained spectrum is shown as close circles and the best fit by CDSSTR [16] is shown by solid line. The normalized root mean square deviation is 0.015.

Next, secondary, tertiary and quaternary structures of MERS-CoV PL^pro^ were investigated, respectively. CD measurement displayed a spectrum which shows negative ellipticity between 240 and 205 nm and positive between 205 and 190 nm (Figure 2). After analyzed by CDSSTR method [16], the best-fit result showed that MERS-CoV PL^pro^ has 23% of α-helix, 31% of β-sheet, and 46% of random coil. The consist is close to that of SARS-CoV PL^pro^ (pdb code: 4M0W) by X-ray crystallography, which has 26% of α-helix, 36% of β-sheet, and 38% of random coil [12]. It suggests that both PL^pro^ may have a similar scaffold.

**Figure 3:**
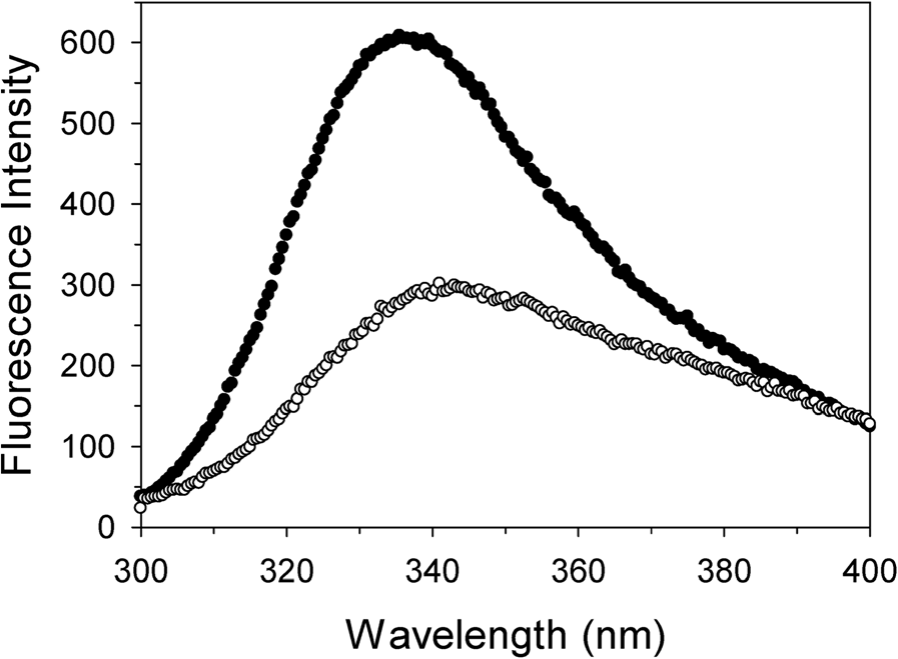
**Fluorescence spectrum of MERS**-**CoV PL**^**pro**^**.** The protein of 1 μM was dissolved in 50 mM phosphate pH 6.5 (closed circles) or 9 M urea (open circles) and excited with 280 nm UV light. The protein fluorescence emission was monitored from 300 to 400 nm at 25°C.

**Figure 4:**
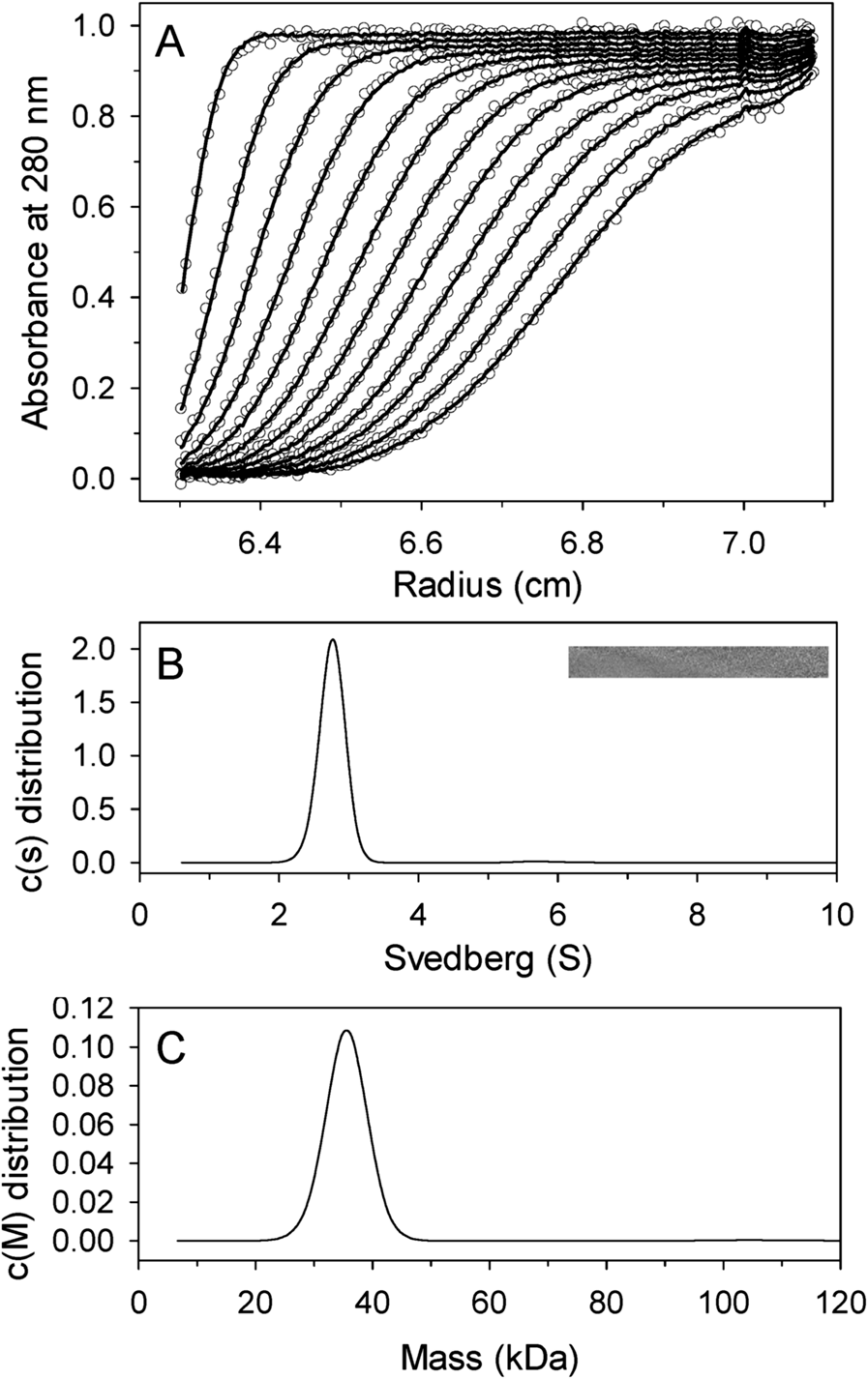
**The continuous size distribution change of MERS-CoV PL**^**pro**^**. (A)** Traces of absorbance at 280 nm of the enzyme in the 50 mM phosphate pH 6.5 during the SV experiment. The protein concentration was 1 mg/ml. For clarity, only every four scan is shown. The symbols represent experimental data and the lines are the results obtained after fitted to the Lamm equation using the SEDFIT program [23,26]. **(B)** and **(C)** show the continuous c(s) and c(M) distribution of PL^pro^, respectively. The residual bitmap of the raw data and the best-fit results are shown in the inset.

The Tyr/Trp fluorescence of MERS-CoV PL^pro^ at the phosphate buffer without or with 9 M urea were also identified (Figure 3). The measurement indicated that the fluorescent intensity of native PL^pro^ (Figure 3, close circles) shows a 70% increase, as compared with that of the denatured form in urea (Figure 3, open circles). On the other hand, the fluorescence emission spectrum of the native MERS-CoV PL^pro^ shows a maximum at 336 nm, while that of the unfolded one shifts to 340 nm. The tendency is similar to that of SARS-CoV PL^pro ^[19] and suggests a folded structure. Next, we also performed AUC experiments to characterize the quaternary structure of MERS-CoV PL^pro^. Figure 4A shows a typical absorbance trace at 280 nm of the PL^pro^ during the experiment. After fitting the signals to a continuous size-distribution model, it was clear that the PL^pro^ was monomeric with a sedimentation coefficient of 2.8 S and molar mass of 35.5 kDa (Figure 4B and Figure 4C), consistent with that for SARS-CoV PL^pro ^[12,19]. All of these biophysical observation confirmed that the PL^pro^ of MERS-CoV and SARS-CoV should have a very similar structure; albeit they only show 30% sequence identity and 50% similarity [12]. Recent studies hypothesized that the homology model of MERS-CoV PL^pro^, like other coronaviral PL^pro^, is a right-hand-like architecture consisting of palm, thumb and fingers domains [10,25].

### Proteolytic activity of MERS-CoV PL^pro^

**Figure 5:**
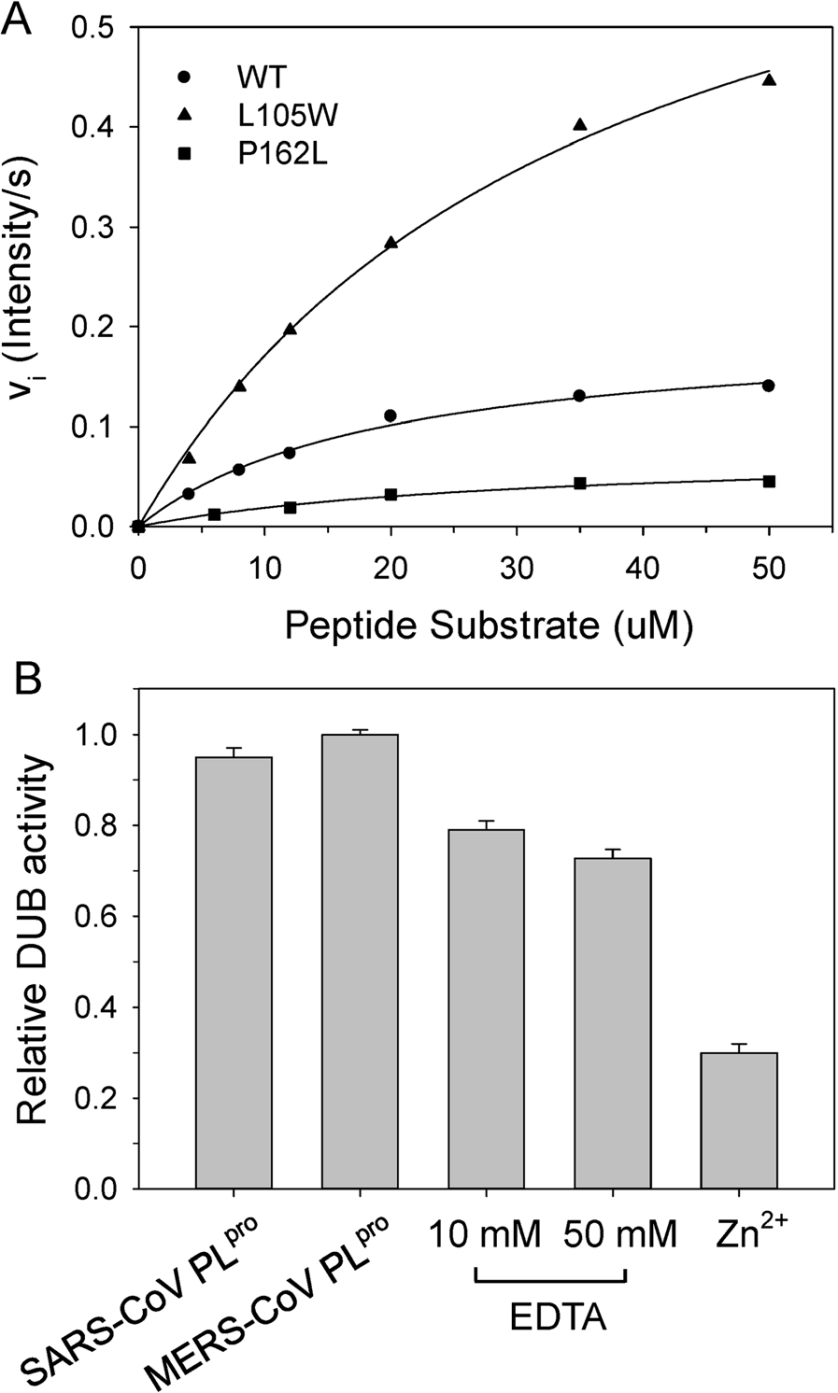
**Proteolytic and DUB activity assay of MERS-CoV PL**^**pro **^**and its mutants.** Panel **(A)** shows the plot of initial velocities versus the concentration of peptidyl substrate, Dabcyl-FRLKGGAPIKGV-Edans. The concentration of the wild-type MERS-CoV PL^pro^ (by circles), the L105W (by triangles) and P162L mutants (by squares) was 1, 0.1 and 10 μM, respectively. The line represented the best-fit results according to the Michaelis-Menten equation (Eq. 1). The kinetic parameters derived are shown in Table 2. **(B)** DUB activity analysis. The fluorogenic substrate Ub-AFC (1 μM) was used as the substrate. For comparison, both DUB activity of SARS-CoV and MERS-CoV PL^pro^ was tested. The protein concentration was 0.17 μM. Besides, the inhibition of MERS-CoV PL^pro^ by 10–50 mM EDTA or 50 μM Zn^2+^ were also clarified.

**Figure 6:**
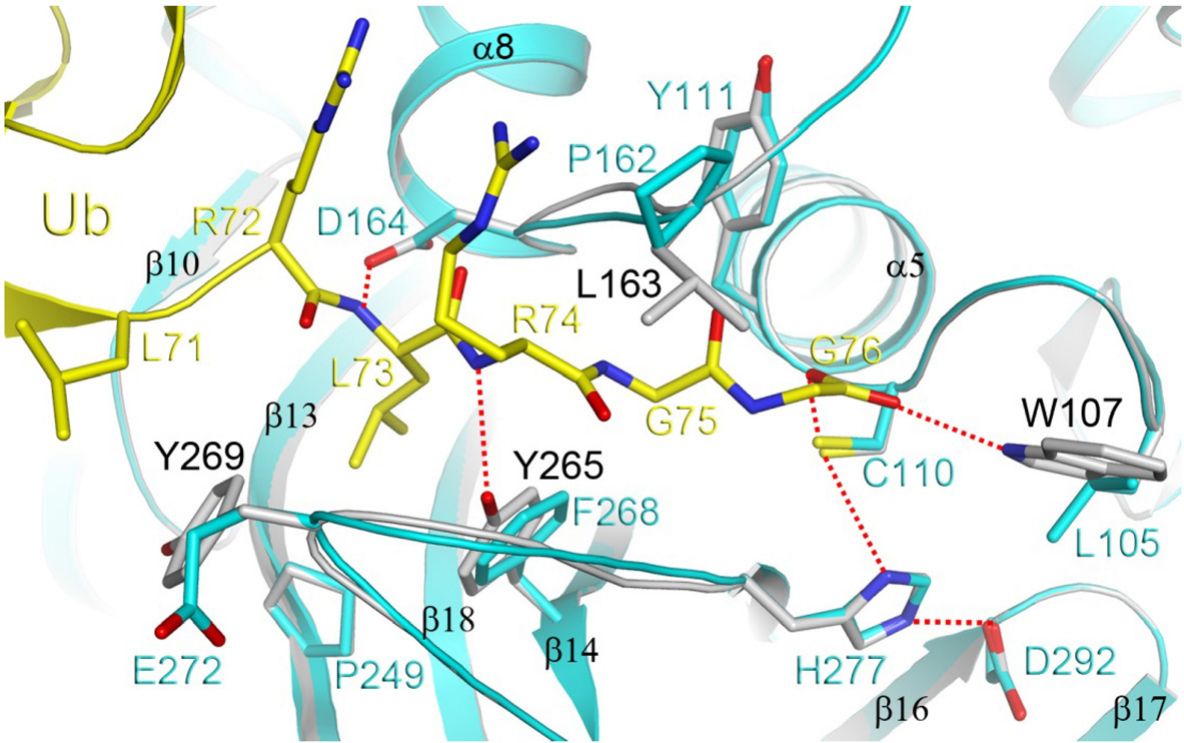
**Putative active site of MERS-CoV PL**^**pro**^**.** The model structure of MERS-CoV PL^pro^ (in cyan) was generated by SWISS-MODEL [27] and then overlaid with the structure of SARS-CoV PL^pro^ (in grey) in complex with Ub (in yellow) (PDB code: 4M0W). The residues are shown as sticks and hydrogen bonding and ion-pair interactions are indicated by red dashed lines. Four residues of SARS-CoV PL^pro^, Trp107, Leu163, Tyr265 and Tyr269, are labeled in black. The figure was produced using PyMol (http://www.pymol.org).

Besides the structural similarity, previous studies have suggested that MERS-CoV PL^pro^ is also a multifunctional enzyme with protease, deubiquitinating and interferon antagonist activities [10]. MERS-CoV PL^pro^ has a catalytic triad which is able to recognize and cleave at LXGG consensus cleavage sites; however, the detail enzyme kinetic mechanism is not known. Here we used the peptidyl substrate, Dabcyl–FRLKGGAPIKGV–Edans, to measure the proteolytic activity of MERS-CoV PL^pro^ (Figure 5A and Table 2). Interestingly, compared with that of SARS-CoV, MERS-CoV PL^pro^ is less active, with a 22-fold loss in k_cat_/K_m_, as a result of a 27.5-fold loss in k_cat_ and 1.3-fold loss in K_m_. According to the sequence alignment and homology modeling, most important residues for the catalysis, including the catalytic triad, Cys110-His277-Asp292 and the residues for substrate P4-P1 binding, Asp164, Pro249, and Gly276 (Asp165, Pro249, and Gly272 in SARS-CoV PL^pro^) are highly conserved (Figure 6) [10,12]. Previous studies have confirmed that Y265F mutant of SARS-CoV PL^pro^ still maintained comparable proteolytic activity with the wild-type [12]. It indicates that the equivalent residue in MERS-CoV PL^pro^, Phe268, is able to make a hydrophobic contact with the substrate P4 residue (Ub-Leu73). Furthermore, although different to the residue Tyr269 of SARS-CoV PL^pro^, the equivalent residue Glu272 of MERS-CoV PL^pro^, whose carboxyl group can point toward outside the hydrophobic pocket, may not interfere the binding of substrate P4 residue (Figure 6). By contrast, as a putative oxyanion bound residue (Tyr107 in SARS-CoV PL^pro^) [15], the equivalent residue Leu105 of MERS-CoV PL^pro^ cannot provide any hydrogen bonding interaction with oxyanion (Figure 6). It will disfavor the formation of tetrahedral intermediate. Otherwise, different to the Leu163 of SARS-CoV PL^pro^, the distinct circular structure of the equivalent residue Pro162 of MERS-CoV PL^pro^ may be too short to hover above the active site for substrate binding and serve to enhance the nucleophilicity of the catalytic triad residue, Cys110 (Figure 6). These two point mutations in MERS-CoV PL^pro^ may significantly lower the catalytic efficiency.

**Table 2:** **The kinetic parameters and DUB activity of MERS**-**CoV PL**^**pro**^

**Proteins**	**Peptide cleavage**			**Deubiquitination**
	**K**_**m**_	***k***_**cat**_	***k***_**cat**_/**K**_**m**_	**Activity (Intensity**/**s)**^**b**^
	**(μM)**^**a**^	**(10**^-**2**^ **s**^-**1**^**)**^**a**^	**(10**^-**3**^ **s**^-**1**^ **μM**^-**1**^**)**	
MERS-CoV PL^pro^				
Wild-type	19.2 ± 2.6	0.4 ± 0.02	0.2 ± 0.03	0.11 ± 0.02
L105W mutant	35.7 ± 3.8	16.5 ± 0.9	4.6 ± 0.6	0.11 ± 0.01
P162L mutant	30.8 ± 8.0	0.01 ± 0.001	0.003 ± 0.001	0.004 ± 0.001
SARS-CoV PL^pro^	25.2 ± 5.1^c^	11 ± 2^c^	4.4 ± 1.2^c^	0.12 ± 0.02

^a^Kinetic data of MERS-CoV PL^pro^ and its mutants were fitted to the Michaelis-Menten equation (Eq. 1). The R_sqr_ were from 0.986 to 0.997, respectively. All the assays were repeated several times to ensure reproducibility.

^b^Fixed concentrations of Ub-AFC (0.5 μM) and PL^pro^ (0.17 μM) were used.

^c^The values were from our previous studies [24].

To verify this, we produced the L105W and P162L mutants of MERS-CoV PL^pro^, and our kinetic data showed that the L105W mutant has a 23-fold increase in activity measured based on k_cat_/K_m_, as a result of a 41-fold increase in k_cat_ and 1.9-fold increase in K_m_ (Figure 5A and Table 2). The results conform to our prediction. However, in contrast, the P162L mutant has a 67-fold loss in k_cat_/K_m_, as a result of a 40-fold loss in k_cat_ and 1.6-fold increase in K_m_ (Figure 5A and Table 2). It suggests the requirement of the Proline residue in this site, although the reason is still not known. Nevertheless, the significant activity recovery by L105W mutation confirms the essential role of this residue on coronaviral PL^pro^ catalysis. Theoretically, PL^pro^ with lower proteolytic activity may result in late maturation of viral nsp1, nsp2, and nsp3 proteins; nonetheless, its influence on MERS-CoV remains unknown.

### DUB activity of MERS-CoV PL^pro^

To characterize the DUB activity of MERS-CoV PL^pro^, the fluorogenic substrate Ub-AFC was used. Interestingly, in contrast with its rather low proteolytic activity, MERS-CoV PL^pro^ shows comparable DUB activity to SARS-CoV PL^pro^ (Table 2 and Figure 5B). It suggests that the two PL^pro^ may show similar binding ability to the Ub core domain (residue 1–72). However, it is inconsistent with our previous observation on the structure of SARS-CoV PL^pro^ in complex with Ub [12]. As mimicking the equivalent residue of MERS-CoV PL^pro^, the arginine mutation of a key residue for Ub core domain binding, Glu168, can result in unstable binding of SARS-CoV PL^pro^ and Ub and significant loss of DUB activity [12]. To verify this inconsistency, a structure of MERS-CoV PL^pro^ in complex with Ub is quite necessary.

Structural characterization of type 1 and type 2 PL^pro^ have revealed that there are four cysteine residues coordinating to a zinc ion within the fingertips region in the finger domain [25,28]. Remove of zinc from SARS-CoV PL^pro^ will cause the tertiary structure more unstable and lead to less active [19]. Based on sequence alignment, MERS-CoV PL^pro^ also has four cysteine residues (Cys190, Cys193, Cys225 and Cys227) on the corresponding position. Here the DUB activity of MERS-CoV PL^pro^ in various EDTA was examined to delineate the possible metal ion effect. The activity was 79% in 10 mM, and 72% left in 50 mM EDTA (Figure 5B). These results suggest the existence of endogenous metal ion, which is beneficial for its DUB activity. By the way, it has been clarified that exogenous zinc ion can efficiently inhibit SARS-CoV PL^pro^ with the IC_50_ value of 1.3 μM [24,29]. Here we also confirmed the potent inhibitory effect of zinc ion on MERS-CoV PL^pro^ (Figure 5B); whereas the mechanism of this inhibition by zinc is not yet understood.

## Conclusions

In summary, following our protocol, active MERS-CoV PL^pro^ can be expressed by *E. coli* and purified with high yield and high purity. The secondary, tertiary and quaternary structural studies concluded that MERS-CoV PL^pro^ has a similar scaffold to other coronaviral PL^pro^, as a right-hand-like architecture consisting of palm, thumb and fingers domains. The result of functional assay indicated that MERS-CoV PL^pro^ exhibits potent DUB activity but rather low proteolytic activity. A natural mutation, Leu105, is the major reason for this difference. The present study not only demonstrates the structural and functional characterization of MERS-CoV PL^pro^, but provides a foundation for further understanding the coronaviral PL^pro^ family, which is an ideal antiviral target. Next, with pure protein and effective proteolytic activity assay, potent inhibitors of MERS-CoV PL^pro^ can be high throughput screened and identified.

## Abbreviations

^1^AFC: 7-amino-4-trifluoro–methylcoumarin; AUC: analytical ultracentrifugation; β-ME: β-mercaptoethanol; CD: circular dichroism; CoV: coronavirus; DUB: deubiquitinating protease; IRF3: interferon regulatory factor 3; MERS-CoV: Middle East respiratory syndrome coronavirus; PCR: polymerase chain reaction; PL^pro^: papain-like protease; SARS-CoV: severe acute respiratory syndrome coronavirus; SV: sedimentation velocity; Ub: ubiquitin.

## Competing interests

The authors declare that they have no competing interests.

## Authors’ contributions

MHL carried out most experiments and analyzed the kinetic data. SJC expressed and purified the protein. CCC and CHL acquired and analyzed the data by mass spectrometry. SCC amplified the cDNA and constructed the expression plasmid. KWC participated in experimental design on structural analysis. CYS and CYC conceived the whole study, participated in experimental design and wrote the manuscript. All authors read and approved the final manuscript.

## Supplementary Material

Additional file 1: Figure S1 Mass spectrometry of trypsin-digested peptides of the recombinant MERS-CoV PLpro protein. The red peaks show the signals of the peptides with correct mass, while the blue ones show the signals of the peptides with oxidation. X-axis indicates the m/z ratio and Y-axis shows the absorbance intensity. Click here for file
