# Supplementary material for: Structural and functional characterization of MERS coronavirus papain-like protease
Source: J Biomed Sci. 2014 Jun 4;21(1):54. doi: 10.1186/1423-0127-21-54 (PMC4051379; doi:10.1186/1423-0127-21-54)
Supplement: Additional file 1: Figure S1 — Mass spectrometry of trypsin-digested peptides of the recombinant MERS-CoV PLpro protein. The red peaks show the signals of the peptides with correct mass, while the blue ones show the signals of the peptides with oxidation. X-axis indicates the m/z ratio and Y-axis shows the absorbance intensity. [file 1423-0127-21-54-S1.pdf]

**Supplementary materials**

# Structural and functional characterization of MERS coronavirus papain-like protease

Min-Han Lin[1], Shang-Ju Chuang[1], Chiao-Che Chen[1], Shu-Chun Cheng[1], Kai-Wen Cheng[1], Chao-Hsiung Lin[1], Chiao-Yin Sun[2,*], and Chi-Yuan Chou[1,*]

[1]Department of Life Sciences and Institute of Genome Sciences, National Yang-Ming University, Taipei 112, Taiwan

[2]Department of Nephrology, Chang-Gung Memorial Hospital, Keelung 204, Taiwan

[*]Correspondence information for Dr. Chi-Yuan Chou, 155 Li-Nong St., Sec. 2, Taipei 112, Taiwan, R.O.C., Phone: +886-2-28267168, FAX: +886-2-28202449, E-mail: cychou@ym.edu.tw and Dr. Chiao-Yin Sun, 222, Mai-Chin Rd., Keelung 204, Taiwan, R. O. C., Phone: +886-2-24313131 ext. 3170, FAX: +886-2-24335342, E-mail: fish3970@gmail.com

One supplemental figure included.

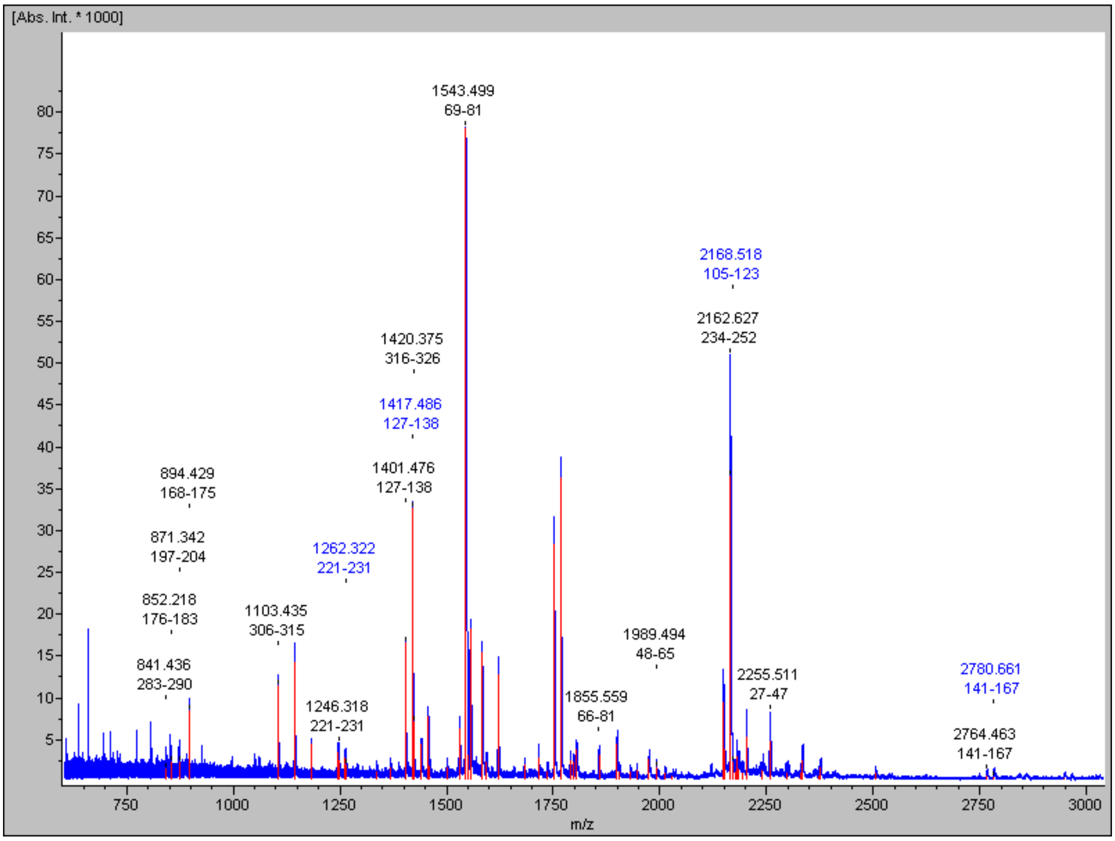


**Supplement Figure. Mass spectrometry of trypsin-digested peptides of the recombinant MERS-CoV $PL^{pro}$ protein.** The red peaks show the signals of the peptides with correct mass, while the blue ones show the signals of the peptides with oxidation. X-axis indicates the m/z ratio and Y-axis shows the absorbance intensity.
